# Bacteriological cure rate and changes in milk composition in mastitis vaccinated ewes affected with subclinical mastitis

**DOI:** 10.14202/vetworld.2018.125-129

**Published:** 2018-02-06

**Authors:** Myassar O. Alekish, Z. Bani Ismail, H. M. Hammouri, M. H. Daradka, S. Al Taha, I. Olymat

**Affiliations:** 1 Department of Veterinary Clinical Sciences, Faculty of Veterinary Medicine, Jordan University of Science and Technology, Irbid 22110, Jordan; 2 Department of Mathematics and Statistics, Faculty of Sciences and Art, Jordan University of Science and Technology, Irbid 22110, Jordan

**Keywords:** immunity, mastitis pathogens, prevention, vaccination

## Abstract

**Aim:**

The aim of this study was to investigate the effects of using a commercially-available polyvalent mastitis vaccine on the bacteriological cure rate of existing subclinical mastitis in Awassi sheep.

**Materials and Methods:**

A total of 164 lactating ewes were divided into two main groups according to udder health and milk somatic cell count (SCC): Group 1=normal (N; n=80) and Group 2=subclinical mastitis (SC; n=84). Each group was then subdivided randomly into two treatment groups: N vaccinated (N_vax_; n=38), N non-vaccinated (N_nvax_; n=42), SC vaccinated (SC_vax_; n=42), and SC non-vaccinated (SC_nvax_; n=42). The vaccine was administered as per manufacturer’s recommendations. Milk samples were collected aseptically from all ewes before vaccine administration (T0) and again on days 28 (T2) and 42 (T3) of the experiment.

**Results:**

In the SC group, the bacteriological cure rates in vaccinated and non-vaccinated ewes were 76% and 69%, respectively. In N group, the new intramammary infection rates in vaccinated and non-vaccinated ewes were 48% and 50%, respectively. Vaccination of normal ewes resulted in a significant (p<0.05) reduction in bacterial growth rate both at day 28 and day 42 of the study. The prevalence of new intramammary infection rate in N_vax_ ewes on days 28 and 42 was 19% and 20%, respectively. The prevalence of new intramammary infection rate in N_nvax_ group on days 28 and 42 was 33% and 30%, respectively. In SC_vax_ group, the bacterial growth rate on days 28 and 42 was 44% and 35%, respectively. In SC_nvax_ group, the bacterial growth rate on days 28 and 42 was 27% and 32%, respectively. There was no statistically significant effect of vaccination on any of the studied milk composition parameters.

**Conclusions:**

This is a preliminary study that indicated a possible protective effect of vaccination against mastitis in sheep. Further, case-controlled studies are indicated to estimate the level of immunity this vaccine provides to vaccinated sheep.

## Introduction

Mastitis is the single most costly disease of dairy animals. Although great technological advances in the prevention and treatment of mastitis have been made in recent years, mastitis continues to cause major economic losses in dairy industry [1,2]. This disease is usually associated with physical and chemical abnormalities of milk through which it can be classified into clinical or subclinical [3,4]. The gold standard diagnostic tool in both clinical and subclinical mastitis is isolation and identification of the causative agent by culture [3,4]. However, California mastitis test, somatic cells count (SCC), and changes in milk constituents are other valuable tools for subclinical mastitis detection [5].

Decreasing the exposure of the udder to potential pathogens and/or increasing the immune response of dairy animals against infection remain some of the most effective mastitis control measures today [6-8]. There have been several research studies that proved the effectiveness of vaccination programs with a different combination of agents against mastitis in dairy cattle and sheep [7,9-11]. Unfortunately, most of the mastitis vaccines are only labeled for dairy cattle. This study is considered the first to investigate the effects of a commercially available polyvalent vaccine in Awassi sheep.

The aim of this study was to investigate the effects of using a commercially-available polyvalent mastitis vaccine on the bacteriological cure rate of existing subclinical mastitis in Awassi sheep.

## Materials and Methods

### Ethical approval

This is to declare that the Animal Care and Use Committee at Jordan University of Science and Technology has approved that all adequate measures were taken to minimize pain or discomfort to all the animals included in this study. The experiment was carried out in accordance with the Guidelines laid down by the International Animal Ethics Committee and in accordance with local laws and regulations.

### Animals and study design

A sheep flock of 164 lactating ewes in their 1^st^ month of lactation located in Al-Mafraq Governorate of Jordan were used in this study. The age of ewes ranged between 1.5 and 5 years. All animals involved in the study were subjected to an initial complete physical examination to determine their general health status. Clinically abnormal sheep were not included in the study. All enrolled sheep received anthelmintic treatment before the start of the study using Ivermectin (Durvet, Missouri, USA) at 0.2 mg/kg subcutaneously twice at 2 weeks interval. Animals were group housed and were supplemented with barley-based diet twice a day and good quality hay and fresh water provided *ad libitum*. Animals were milked by hand twice per day. Pre-dipping of teats before and after milking using iodine-based disinfectant was used routinely in this herd.

To complete the physical examination, both halves of the udder were palpated and any abnormal findings such as swelling, pain, or hotness were recorded. Ewes with clinical mastitis were excluded from the study. Milk secretion was also examined for abnormal color, contents, or consistency.

According to the clinical status of the udder and SCC of milk, ewes were divided into two main groups: Group 1=normal (N; n=80) and Group 2=subclinical mastitis (SC; n=84). Subclinical mastitis was defined as one or both udder halves having a SCC ≥250,000 cells/ml. Each group was then subdivided into two subgroups according to vaccination status: Normal vaccinated (N_vax_; n=38), normal non-vaccinated (N_nvax_; n=42), subclinical mastitis vaccinated (SC_vax_; n=42), and subclinical mastitis non-vaccinated (SC_nvax_; n=42).

### Vaccine administration

A commercially available polyvalent vaccine against *Streptococcus agalactiae*, *Streptococcus dysgalactiae*, *Streptococcus uberis*, *Streptococcus pyogenes*, *Staphylococcus aureus*, *Escherichia coli*, and *Trueperella pyogenes* was used (Mastivac, Ovejero Laboratories, Spain). Each ml of the vaccine contained 6×10^9^ of microorganisms. The vaccine is labeled for use in dairy cattle for the prevention of clinical and subclinical mastitis. The recommended dose in cows is 5 ml administered subcutaneously in the neck region twice at 15 days interval. Ewes in the vaccinated subgroups were administered 3 ml of the vaccine subcutaneously in the neck region on days 7 and 21 of the study. In the non-vaccinated groups, each ewe received 3 ml of sterile normal saline subcutaneously in the neck region on days 7 and 21 of the study.

Ewes were monitored immediately after vaccine administration (heart rate, respiration rate, rectal temperature, and any other abnormal signs). Ewes remained under close observation for 48 h following vaccination. At the time of second vaccine administration, ewes were re-examined and the site of the first dose was palpated to detect swellings, pain, or pus. Sheep were kept under monitoring after the second dose for 48 h, and the site of the second dose was re-examined after 7 days to detect abnormalities.

### Milk sample collection

Milk samples were collected from each quarter before vaccine administration (T0) and on days 28 and 42 of the experiment. For bacterial culture, composite milk samples were collected aseptically from each ewe according to standard procedures of the International Dairy Federation [12]. Briefly, the teat end was cleaned and disinfected using 70% isopropyl alcohol. The first few streams of milk were discarded after which an approximately 5 ml of milk was collected and placed in a sterile tube. Another approximately 50 ml of milk were collected and placed in a screw top container for determination of SCC and milk composition parameters. The samples were transported to the laboratory on ice and tested within 2-3 h.

### Bacterial isolation and identification

A swab from each milk sample was spread on a blood agar and MacConkey agar plates and incubated at 37°C for 24 h. Suspected positive colonies were subcultured into blood agar plate, MacConkey plate, mannitol salt agar, and DNase media at 37°C for 24 h. Bacterial identification was achieved by morphological characterization of its colonies, gram staining followed by chemical testing using coagulase and catalase tests. Further identification of bacteria subspecies was achieved using various commercially available microtube identification systems such as Microbact staph 12S (Oxoid, catalog number: MB1561, Hampshire, UK) and RapID systems (Thermo Fisher Scientific, catalog number: R8311006, Kansas, USA) according to the manufacturer’s recommendations. Bacterial growth rate was determined for each bacterial species and was defined as the number of colonies grown in the culture plate after 24 h of incubation at 37°C. Bacteriological cure rate was defined as milk samples from which bacteria were initially (T0) isolated, and after vaccination, culture showed no growth. New IMI was defined as a sample from which bacteria was cultured for the first time during T2 or T3 (no growth at T0).

### SCC determination

Milk samples for SCC determination were collected before vaccine administration (T0) and on days 28 and 42 of the experiment. SCC was determined by spreading 0.01 ml of gently mixed milk from each sample over 1 cm^2^ area of a glass slide and staining using Newman-Lampert stain. The stained slides were then examined by the same technician every time using light microscope according to previously published procedure [13]. SCC was expressed in log_2_.

### Milk component analysis

Milk samples for analysis of various milk components were collected before vaccine administration (T0) and on days 28 and 42 of the experiment. The following parameters were determined automatically: Milk fat, lactose, crude protein (CP), and solids-non-fat (SNF) (Milko Scope Julie C8; Scope Electric, Regensburg, Germany) according to the manufacturer’s instructions.

### Statistical analysis

Q-Q plots were used to assess normality and apply log transformation of somatic cells in milk samples. Pearson Chi-square test was used to determine possible associations between vaccine and cure rates in the subclinical group as well as new mastitis rates in normal ewes. A repeated measures ANOVA test was used to assess milk composition variables over different sampling points in vaccinated and non-vaccinated normal ewes. p<0.05 was considered statistically significant.

## Results

There were no immediate systemic side effects noticed after vaccine administration. There were also no local vaccine-related swellings at the site of injection noticed at any time during the study period.

In the SC group, the bacteriological cure rates in vaccinated and non-vaccinated ewes were 76% and 69%, respectively (Table-1).

**Table-1:** Bacteriological cure rates in vaccinated and nonvaccinated ewes with subclinical mastitis and new intramammary infection rates in normal ewes following vaccination using a commercially available polyvalent mastitis vaccine.

Ewes with subclinical mastitis	Vaccinated N=25 (%)	Nonvaccinated N=29 (%)	p value
Bacteriological cure rate	19 (76)	20 (69)	0.565

**Normal ewes**	**Vaccinated N=29 (%)**	**Nonvaccinated N=24 (%)**	**p value**

New intramammary infection	14 (48)	12 (50)	0.415

In N group, the new IMI rates in vaccinated and non-vaccinated ewes were 48% and 50%, respectively (Table-1).

Vaccination of normal ewes resulted in a significant (p<0.05) reduction in bacterial growth rate both at day 28 and day 42 of the study (Table-2). The prevalence of new IMI rate in N_vax_ ewes on days 28 and 42 was 19% and 20%, respectively. The prevalence of new IMI rate in N_nvax_ group on days 28 and 42 was 33% and 30%, respectively.

**Table-2:** Effects of vaccination on milk bacterial growth rates in ewes affected with subclinical mastitis and in normal ewes.

Day	Ewes with subclinical mastitis		Normal ewes	
	
	Vaccinated (n=38)	Non-vaccinated (n=42)	Vaccinated ( n=38)	Non-vaccinated (n=42)
0	13	12	8	5
28	44*	27*	19*	33*
42	35	32	20*	30*

* Between rows indicate P<0.05

In SC_vax_ group, the bacterial growth rate on days 28 and 42 was 44% and 35%, respectively (Table-2). In SC_nvax_ group, the bacterial growth rate on days 28 and 42 was 27% and 32%, respectively.

There were no significant effects of vaccination on any of the studied milk composition parameters in normal ewes at any sampling point during the study (Table-3).

**Table-3:** Percentages of various milk composition parameters in vaccinated and Non-vaccinated normal ewes.

Parameters	Vaccinated ewes			Non-vaccinated ewes		
	
	Day 0	Day 28	Day 42	Day 0	Day 28	Day 42
Fat	6.9	6.0	6.5	7.1	7.1	8.5
Solid-non-fat	9.9	10.0	10.8	9.1	10.7	11.5
Crude protein	2.2	3.7	3.9	2.5	3.9	4.2
Lactose	3.0	5.5	5.9	2.4	5.9	6.3

## Discussion

In this study, all vaccinated ewes tolerated the vaccine very well. There were no immediate or late-occurring systemic side effects in any of the vaccinated ewes. These results are similar to those reported previously by others in cows [6]. Although, local vaccine-related swellings have been reported in previous studies, none were reported in this study [6].

Results of the bacteriological cultures in this study indicate a significant effect of vaccination on the growth rate of bacteria from milk samples. However, this effect was non-significant on the rate of new mastitis in normal ewes although a trend toward prevention was evident. Bacterial growth rate was, especially, reduced in normal ewes while this effect was limited in already infected sheep. This may indicate that the vaccine might have provided better protection of healthy udders. These results were also substantiated by the trend of more new mastitis rates in non-vaccinated normal ewes when compared to those that were vaccinated.

Previous studies have failed to show that vaccination reduced new IMI rate due to staphylococci or to provide sufficient antibodies in milk that eliminates staphylococcal bacteria from the mammary gland [14,15]. Other researchers have also concluded that while vaccination may not completely prevent new IMIs, it may result in a substantial decrease in the duration, severity, and transmissibility of the infection [11,16].

Subclinical mastitis in ewes can be detected by performing SCC in fresh milk samples and is considered a sensitive indicator of udder infection [17-19]. In this study, SCC decreased significantly in subclinical mastitis after vaccination while it remained consistently high in the non-vaccinated ewes. This is an important finding which may indicate a substantial decrease of subclinical mastitis incidence rate in vaccinated sheep. These findings are in agreement with those reported previously in cows using a vaccine against *S. aureus* [6,19,20]. This finding was even more fortified by a significantly less count of somatic cells in vaccinated normal ewes.

In this study, although both vaccinated and non-vaccinated normal ewes showed a trend toward increased SCC as the study went along, the increase in SCC was far less significant in the vaccinated ewes which may indicate a significant level of protection provided by the vaccine in this group. These results are similar to those reported previously in sheep [7] and cows [14].

The effects of vaccination on various milk composition parameters (milk fat, SNF, lactose, and CP) in Awassi sheep are being reported here for the first time. Various milk composition parameters were studied, and there was no obvious significant effect on any of these parameters.

In conclusion, results of this study indicate a potential beneficial effect of vaccination against mastitis in lactating Awassi ewes. This effect was evident by a non-significant increase in mastitis cure rate in subclinical mastitis ewes and a decreased rate of new mastitis rates in normal ewes. Further, clinical trials involving pathogen challenge studies in vaccinated and non-vaccinated ewes are warranted to verify the degree of protection this vaccine may provide to lactating sheep.

## Authors’ Contributions

MOA and MHD designed, coor­dinated and supervised the study. ZBI carried out data interpretation and paper writing. HMH performed the statistical analysis. SAT and IO carried out sample collection and laboratory work. All authors read and approved the final manuscript.
